# A century of precipitation trends in forest lands of the Lower Mississippi River Alluvial Valley

**DOI:** 10.1038/s41598-020-69508-8

**Published:** 2020-07-30

**Authors:** Ying Ouyang, Jiaen Zhang, Gary Feng, Yongshan Wan, Theodor D. Leininger

**Affiliations:** 1 Center for Bottomland Hardwoods Research, USDA Forest Service, Mississippi State, MS 39762 USA; 2 Department of Ecology, South China Agricultural University, Guangzhou, 510642 China; 3 Crop Science Research Laboratory, USDA Agricultural Research Service, 810 Hwy 12 East, Mississippi State, MS 39762 USA; 4 Center for Environmental Measurement and Modeling, US EPA, 1 Sabine Island Drive, Gulf Breeze, FL 32561 USA; 5 Center for Bottomland Hardwoods Research, USDA Forest Service, 432 Stoneville Road, Stoneville, MS 38776 USA

**Keywords:** Climate sciences, Hydrology

## Abstract

Variations in long-term precipitation trends due to climate forcings have been observed in many parts of the world, exacerbating hydrological uncertainties to predicting droughts, floods, water resource availability, and ecosystem services. The Lower Mississippi River Alluvial Valley (LMRAV) is an important economic region of the midsouth USA, which is prone to natural disasters from extreme climate events and is known historically for cyclic flooding events and, within the last 20 years, for groundwater level declines. However, our knowledge of long-term precipitation trends in this region is fragmented. Using 100-year historic daily precipitation data from six stations of forest lands along with multivariate statistical analysis, we found that there were significant increasing trends (*p* ≤ 0.05) in annual precipitation near the south coastal area of the LMRAV and only marginally increasing trends in the northern area. Spatial variation in seasonality was observed at the decadal scale with increasing trends in fall near the coastal area and in spring around the north area. In addition to becoming wetter, the coastal area also experienced higher precipitation intensity with shorter return period over the past 100 years. These findings are useful to water resource managers for adapting to changing climate conditions in the LMRAV.

## Introduction

Variations in long-term precipitation trends due to climate forcings have been observed in many parts of USA and around the world^1–7^. Such variations could modify hydrological processes and add uncertainties to predicting droughts, floods, water resources availability, and ecosystem services. It has been reported that the portion of the USA, with the most intensive single-day precipitation events, has increased about one half of 1% per decade from 1910 to 2015^8^. At a regional scale, the Mediterranean and northeastern European regions have been identified as primary climate change hot spots^2^. These changes in regional climate have resulted in increased rainfall in the Northern Hemisphere mid-latitudes, less rainfall in the Northern Hemisphere subtropics and tropics, and more precipitation the Southern Hemisphere subtropics and deep tropics in recent decades^9^. For some given basins, flooding events are directly related to precipitation intensity and duration, which, in turn, depend on synoptic changes, land use alterations, and other microclimatic conditions^10,11^. The temporal precipitation variability with intensified extremes has significant impacts on human health, agricultural production, commercial activity, and ecological services^5^. Predictions by the Intergovernmental Panel on Climate Change (IPCC) indicate that precipitation events are very likely to change in intensity, frequency and location throughout the twenty-first century^3^.

An important factor compounding long-term climate-driven trend of annual precipitation is the naturally occurring “teleconnection pattern” between sea surface temperature anomalies and decadal variability of precipitation. For example, the Atlantic Multidecadal Oscillation (AMO) has been linked to the decadal precipitation fluctuation^12^. McCarty et al.^12^ investigated sea level changes in the east coast of the US associated with decadal ocean circulation. These authors cited that the decadal ocean circulation responds to the cooling and warming cycles of AMO. However, the impact of the AMO cycles on precipitation in the Lower Mississippi River Alluvial Valley (LMRAV) is basically unknown.

**Figure 1:**
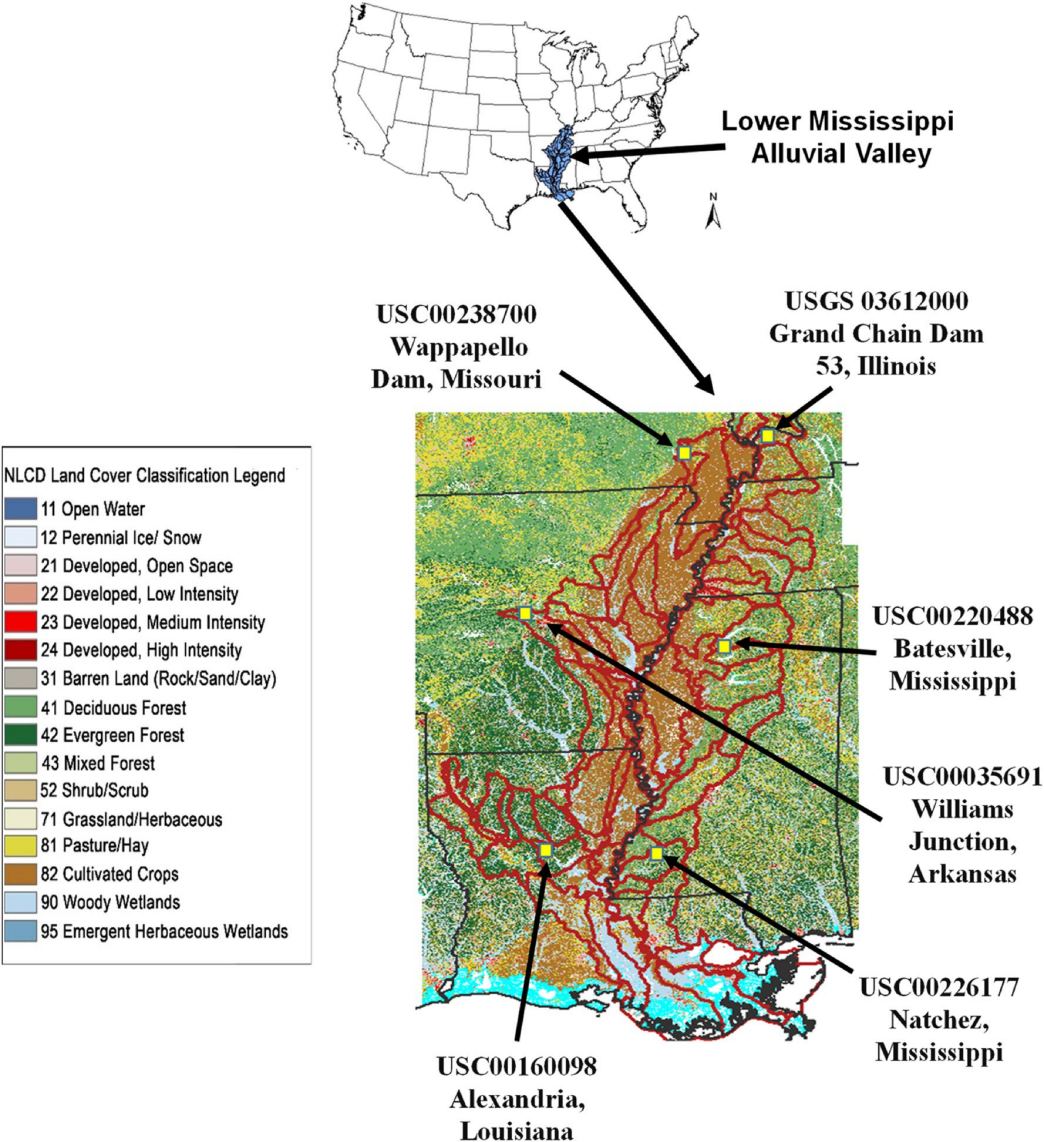
Location map of the Lower Mississippi River Alluvial Valley (LMRAV) showing the six weather stations in forest lands used in this study, which is created using ArcGIS Version 10.5.1.

The LMRAV, an intensive agricultural and forest production region and an important economic region in midsouth USA, is located in the floodplain of the Mississippi River (MR) starting at Illinois and continuing through Missouri, Kentucky, Arkansas, Tennessee, Mississippi, and Louisiana^13^ (Fig. 1). Land use change and climate variability in the LMRAV, including clearcuttings of bottomland hardwood forests, conversions of forests to agricultural lands and loss of connected floodplain, are largely responsible for river flooding, wetland loss, and water quality degradation in the MR and the adjacent Gulf of Mexico^14–17^. More specifically, the LMRAV is prone to natural disasters from extreme climatic events and is well known for the occurrence of cyclic flooding since 1928^18^. Additionally, the extreme climatic events have degraded surface water quality through flooding, which brings high levels of turbidity, organic matter, and contaminants^19–22^. Climatic extremes associated with substantial economic loss have prompted extensive efforts by federal and state agencies to understand, predict, and manage stream flow, water quality, and river flooding in the LMRAV^11^.

Flow, distribution, and quality of water in forest watersheds are regulated by forests^25^. Among the many ecological functions provided by forests, they also process water used for agriculture, industry, and human consumption^23^. Changes in long-term trends of precipitation due to climate change would modify hydrologic processes and increase the uncertainty of these processes occurring in forest watersheds. Therefore, an assessment of these trends is essential to water resource management, water supply planning, environmental protection, and ecological services in forest watersheds. In recent years, several studies have investigated the impacts of climate change on hydrologic processes in the forest watersheds, including stream discharge, overland flow, surface evaporation, and water yield^24–30^. Ouyang et al.^16^ studied the impacts of projected rainfall and air temperature variations upon water discharge, water yield, and evaporative loss in the Lower Yazoo River Watershed, which is a sub-basin in the LMRAV. They found that a 6.4% decrease in rainfall amount results in 11.8% and 10.3% decreases in water yield and evaporative loss, respectively. Dyer and Mercer^4^ assessed spatial variability of rainfall in the LMRAV using rotated principal component analysis with radar-derived rainfall data from 1996 to 2011. These authors found that although there is substantial variability among seasons, the precipitation depths in the region are generally lower with more variations than the eastern areas of the LMRAV. They further argued that a warm season rainfall volume is generally lower and less predictable in the LMRAV as compared to a cool season. Currently, few studies have assessed the long-term trends of precipitation in forest watersheds of the LMRAV.

Improving flood forecasting, water resource management, and water quality protection for the LMRAV depends on understanding the linkages between precipitation trends and hydrological processes. Since an individual precipitation event cannot be simply and directly attributed to climate change impacts, long-term trends of precipitation such as duration, frequency and intensity, need to be analyzed at several localities in a region to identify any climate change impacts. The goal of this study was to assess trends in the past 100 years, approximately from 1900 to 2018, of precipitation data in six headwater forest watersheds of the LMRAV using multivariate statistical analysis. Our specific objectives were to: (1) identify the relationship between precipitation intensity and its recurrence interval, (2) estimate the annual and decadal seasonal trends of precipitation, and (3) assess the impact of AMO on precipitation in the LMRAV. The forest watersheds selected for this study are upstream of areas with intensive agricultural crop production in the LMRAV. It is important to understand how changing precipitation in forested areas of the world effects crop production downstream. The six headwater forest watersheds were selected because there exists a continuous record of precipitation data for 100 or more years. Forested headwater areas were chosen because of the relative lack of disturbance to these lands over the period of interest, which provides a better opportunity for analyzing how climate change affects precipitation trends.

## Results

### General assessment and recurrence period of precipitation

Comparison of daily precipitations shows that five out of six stations (except for the USC0013580 station in Grand Chain Dam 53, Illinois) had very similar descriptive statistics (Table 1). Among these five stations, the ranges were from 3.12 to 3.98 mm for mean precipitation, from 9.20 to 11.99 mm for standard deviation, from 5.23 to 5.63 for skewness, and from 40.18 to 48.54 for kurtosis. In contrast, the mean precipitation (0.13 mm) and standard deviation (0.19 mm) of the Illinois station (USC00113580) were about one order of magnitude lower but the skew (12.48) and kurtosis (238.94) were much higher than those of other five stations.

**Table 1:** Location, land use, and descriptive statistics of daily precipitation for the six selected NOAA weather stations in forest lands of the LMRAV.

Parameter	USC00238700	USC00113580	USC00035691	USC00220488	USC00160098	USC00226177
Location	Wappapello Dam, Missouri	Grand Chain Dam 53, Illinois	Williams Junction, Arkansas	Batesville, Mississippi	Alexandria, Louisiana	Natchez, Mississippi
Land use	Forest decidous	Forest decidous	Forest evegreen	Forest decidous	Forest evergreen	Forest decidous
Period of record	1918–2018 (101 years)	1901–2018 (118 years)	1900–2018 (119 years)	1900–2016 (117 years)	1900–2018 (119 years)	1901–2017 (117 years)
Mean (mm)	3.94	0.13	3.37	3.66	3.98	3.12
Standard error (mm)	0.06	0.00	0.05	0.05	0.06	0.05
Standard deviation (mm)	11.99	0.91	9.96	10.80	12.20	9.20
Sample variance	143.82	0.82	99.29	116.55	148.81	84.62
Kurtosis	48.54	238.94	52.58	40.18	48.72	40.36
Skewness	5.61	12.48	5.63	5.12	5.63	5.23
Maximum (mm)	266.70	38.35	224.00	224.79	254.00	161.00

**Figure 2:**
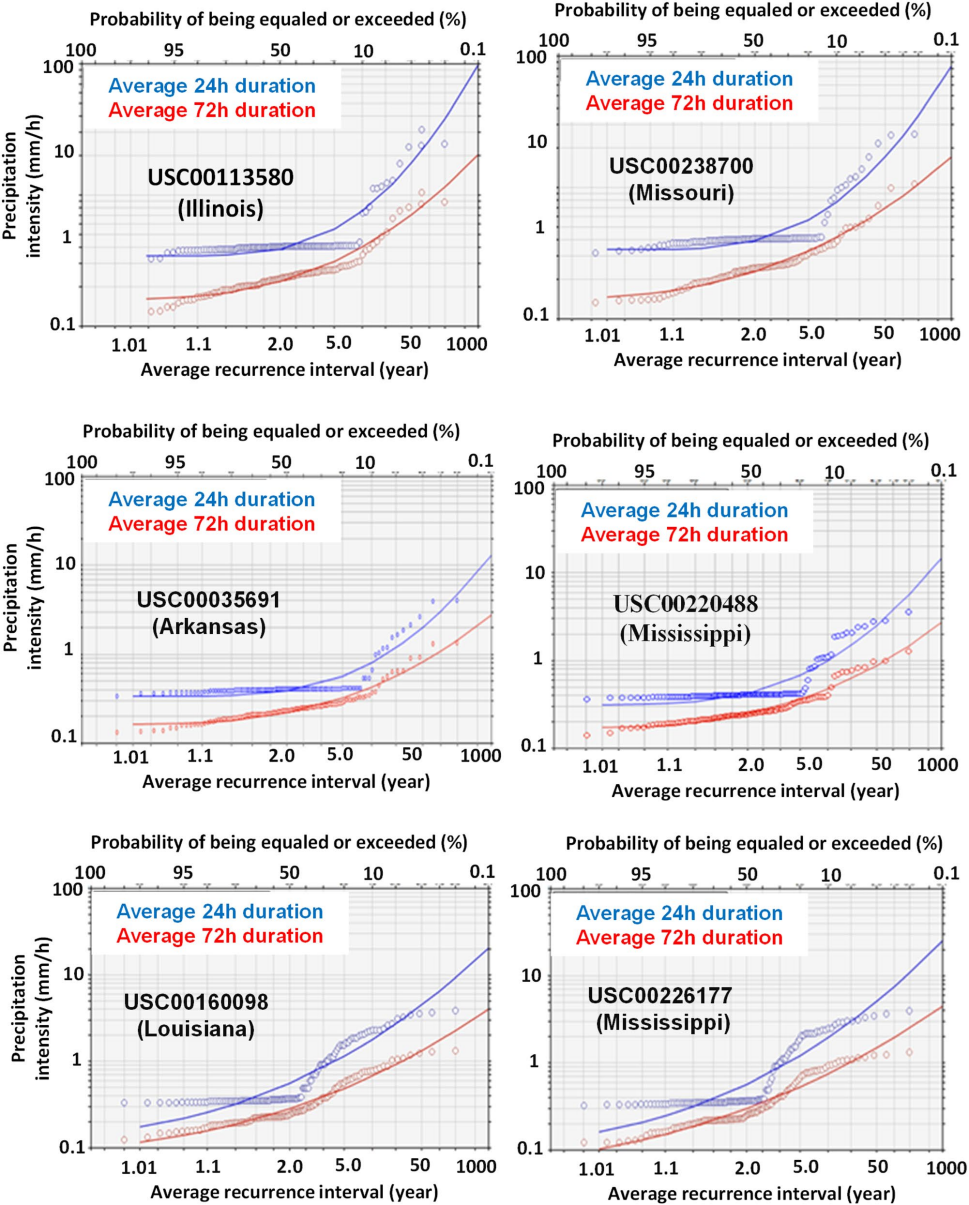
Relationship of precipitation intensity and average recurrence interval at 24- and 72-h durations for the six weather stations used in this study. The probability of precipitation intensity occurred at a given recurrence interval is also shown.

Precipitation intensity and its recurrence interval under different durations are important indicators to extreme precipitation events. The average recurrence intervals (or periods) of precipitation intensity for the six stations are given in Fig. 2. For the average recurrence interval of 2 years at a 24-h duration, the precipitation intensities were 0.51, 0.52, 0.40, 0.39, 0.30, and 0.31 mm/h, respectively, for USC00160098, USC00226177, USC00035691, USC00220488, USC00238700 and USC00113580, and the probability for these intensities to be equaled or exceeded was ≥ 50% (Fig. 2). For the average recurrence interval of 10 years at a 24-h duration, the precipitation intensities were 1.8, 1.1, 0.81, 0.98, 0.75, and 0.78 mm/h, respectively, for USC00160098, USC00226177, USC00035691, USC00220488, USC00238700, and USC00113580, and the probability for these intensities to be equaled or exceeded was ≥ 10% (Fig. 2).

### Annual trends

**Figure 3:**
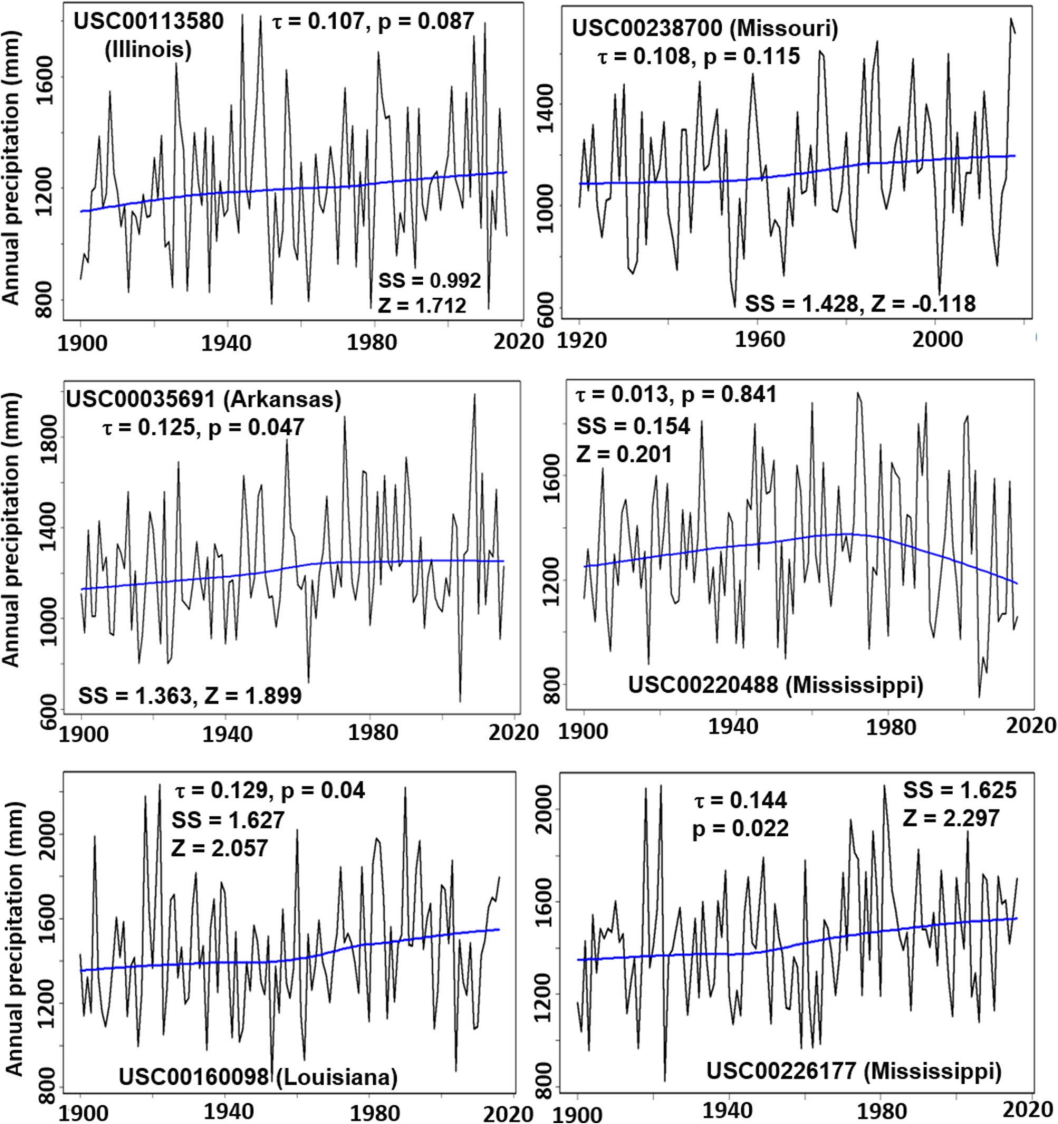
Annual precipitation for the six weather stations with Mann Kendall test results. The smoothing line is drawn using the Local Weighted Scatterplot Smoothing (LOWESS) function. Note that the y-axis scales are different for each sub-plot.

Annual variations in total amounts of precipitation over the past 100 or more years for the six weather stations associated with Mann Kendall’s tau (τ), p value, Sen’s slope (SS), and z score are given in Fig. 3 and Table 2. In Mann Kendall time series statistic, the value τ ranges from − 1 to 1 and is a measure of relationships between variable and time, where 0 is no relationship and 1 (or − 1) is a perfect relationship (with positive τ for increasing trend and negative τ for decreasing trend). The p value is a statistical measure of a trend, and if *p* ≤ 0.05 there is a monotonic trend^31^. The value of SS is used to estimate the magnitude of change in Mann Kendall’s trends, while the value of z-score tells how many standard deviations from the mean^31,32^.

**Table 2:** Mann Kendall statistics of the annual and decadal seasonal precipitations as well as the annual wet days for the NOAA weather stations used in this study.

Parameter	USC00238700 in Wappapello Dam, Missouri	USC00113580 in Grand Chain Dam 53, Illinois	USC00035691 in Williams Junction, Arkansas	USC00220488 in Batesville, Mississippi	USC00160098 in Alexandria, Louisiana	USC00226177 in Natchez, Mississippi
**Annual precipitation**						
Kendall's τ	0.108	0.107	0.125	0.013	0.129	0.144
Sen's slope	1.428	0.992	1.364	0.154	1.627	1.625
*p* value	0.115	0.087	0.047	0.841	0.040	0.022
z score	− 0.118	1.712	1.989	0.201	2.057	2.297
**Decadal seasonal precipitation**						
Kendall's τ (Spring)	0.556	0.556	− 0.152	− 0.091	− 0.236	0.055
Sen's slope (Spring)	62.075	66.403	− 19.750	− 8.250	− 61.686	19.685
*p* value (spring)	0.048	0.013	0.537	0.756	0.350	0.876
z score (Spring)	1.981	2.491	− 0.617	− 0.311	− 0.934	0.156
Kendall's τ (Summer)	− 0.278	− 0.200	0.000	− 0.121	0.091	− 0.127
Sen's slope (Summer)	− 37.667	− 11.853	− 2.823	− 27.070	10.160	− 38.735
*p* value (Summer)	0.348	0.436	1.000	0.631	0.756	0.640
z score (Summer)	− 0.938	− 0.779	0.000	− 0.480	0.311	2.647
Kendall's τ (Fall)	0.333	0.200	0.212	0.527	0.600	0.636
Sen's slope (Fall)	54.017	42.545	58.833	112.333	128.693	148.449
*p* value (Fall)	0.252	0.436	0.373	0.029	0.013	0.008
z score (Fall)	1.147	0.779	0.891	2.180	2.491	2.647
Kendall's τ (Winter)	0.667	0.127	0.587	− 0.236	0.127	0.127
Sen's slope (Winter)	79.243	15.240	190.000	− 2.083	25.400	30.480
*p* value (Winter)	0.016	0.640	0.015	0.350	0.640	0.640
z score (Winter)	2.398	0.467	2.421	− 0.934	0.467	0.467
**Annual wet day**						
Kendall's τ	0.333	0.260	− 0.215	− 0.236	0.443	0.636
Sen's slope	5.310	5.292	− 0.450	− 2.083	0.686	1.717
*p* value	0.592	0.271	0.370	0.350	0.050	0.005
z score	1.100	1.100	− 0.896	− 0.934	1.925	2.812

Figure 3 shows that there were significant (*p* ≤ 0.05) increasing trends in annual precipitation for three (i.e., USC00226177 in Mississippi; USC00160098 in Louisiana; and USC00035691 in Arkansas) out of six weather stations. The first two stations are located in south LMRAV, which are close to the Gulf of Mexico and the last station is situated in the west center of the LMRAV (Fig. 1). The increasing trends were in the following order: USC00160098 (SS = 1.627) > USC00226177 (SS = 1.625) > USC00035691 (SS = 1.364) based on Sen’s slope. These trends had about two-unit deviations above the means as indicated by their z scores. In contrast, no significant trends were observed for the two stations (i.e., USC00113580 in Illinois and USC00238700 in Missouri) located in north (inland) LMRAV and the one station (i.e., USC00220488 in Mississippi) located in east center of the LMRAV (Figs. 1, 3) although they all had the increasing trends (i.e., the positive τ values).

### Decadal wet days

**Figure 4:**
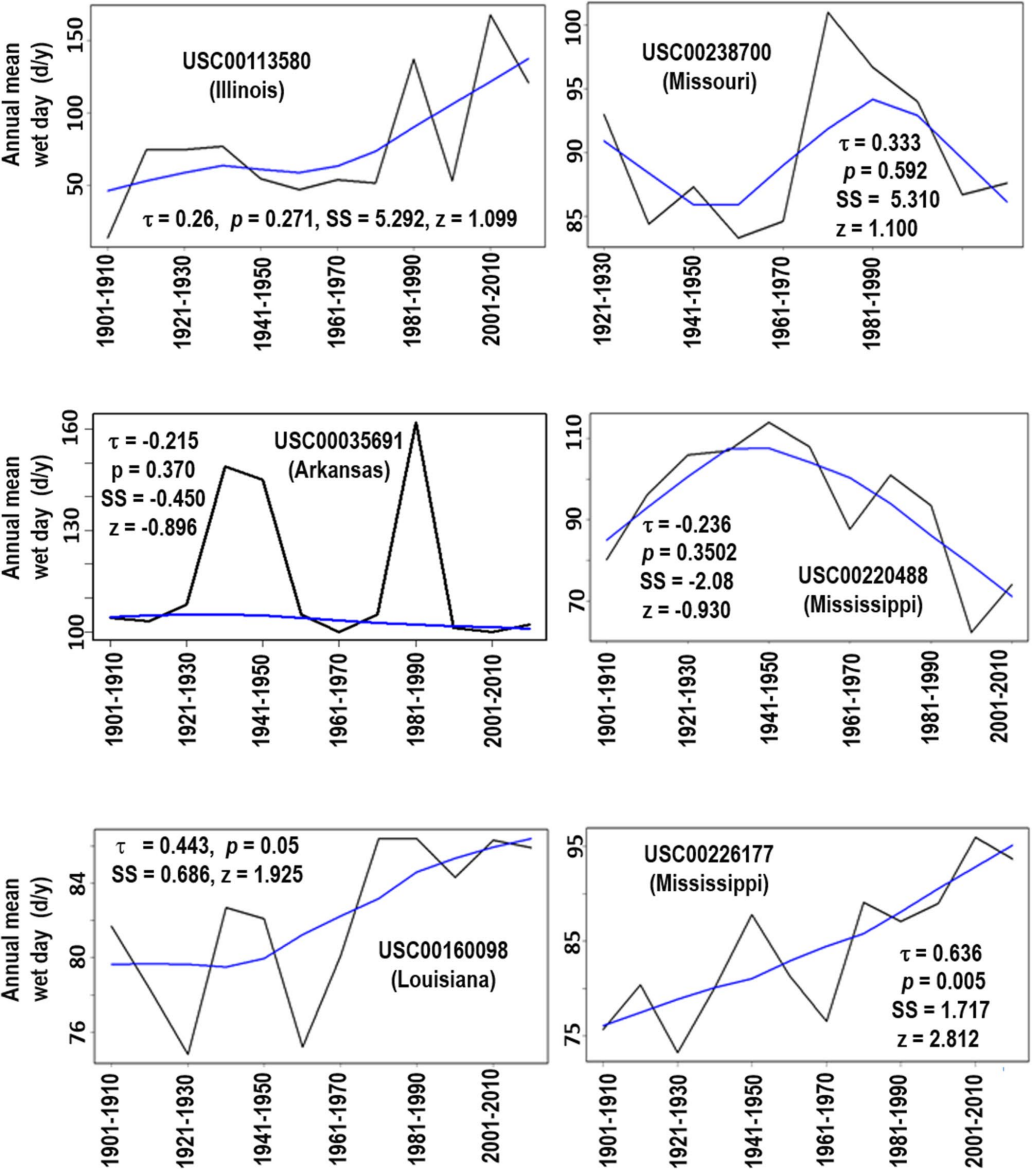
Annual mean wet days in decadal scale for the six weather stations with Mann. Kendall test results. The smoothing line is drawn using the Local Weighted Scatterplot Smoothing (LOWESS) function. Note that the y-axis scales are different for each sub-plot.

Annual mean wet days in decadal scale over the past 100 or more years for the six weather stations are shown in Fig. 4. In this study, a day that has any precipitation was considered as a wet day, and the mean wet days in every decade were obtained using the HYDSTATR (rainfall statistical summary report) function from the HYDSTRA model (Kisters Inc.). Analogous to the case of the total amount of precipitation, there were significant (*p* ≤ 0.05) increasing trends in annual mean wet days at decadal scale for two (i.e., USC00226177 in Mississippi and USC00160098 in Louisiana) out of six weather stations (Fig. 4). The annual mean wet days were 79 and 75.5 d/y from 1901 to 1910 but were 86 and 96 d/y from 2001–2010, respectively, for USC00160098 and USC00226177, actualizing 8 and 27% increase after 100 years. Based on the Sen’s slopes, the rate of the increasing trend of USC00226177 (SS = 1.717) was greater than that of USC00160098 (SS = 0.686). No significant trends in annual mean wet days were observed for the other four stations located in the center and north of the LMRAV (Fig. 4) although stations in the center had the decreasing trends while the stations in the north had the increasing trends.

### Decadal seasonality

**Figure 5:**
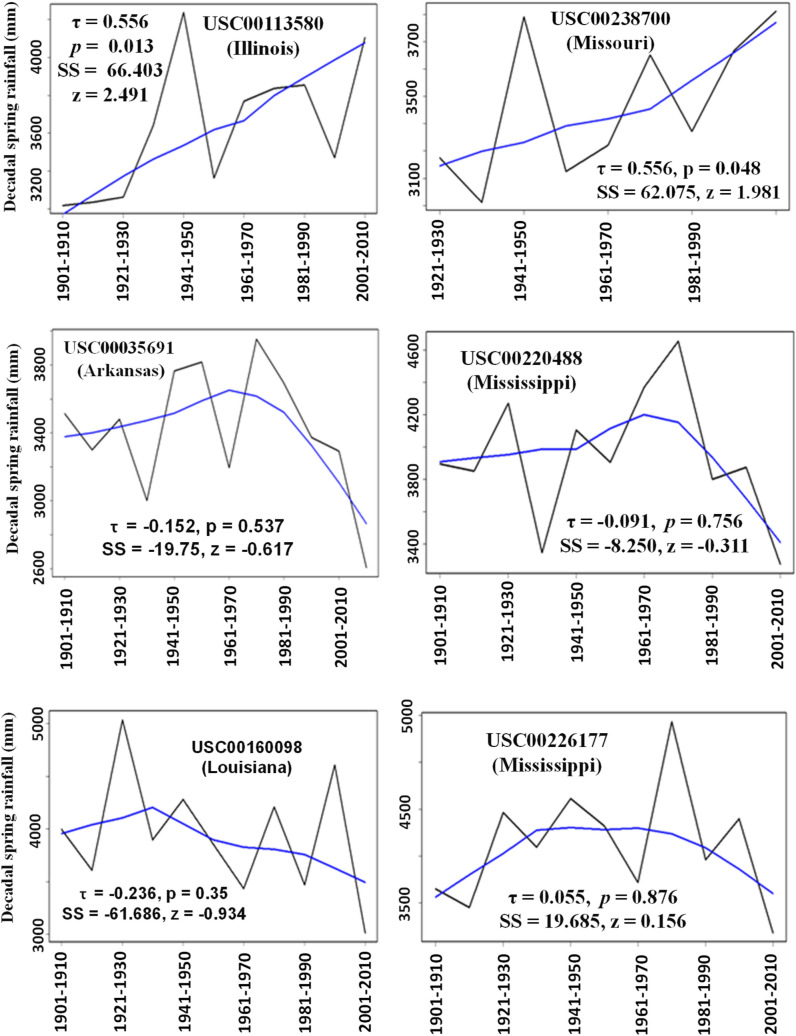
Amount of decadal precipitation in spring season for the six weather stations with Mann Kendall test results. The smoothing line is drawn using the Local Weighted Scatterplot Smoothing (LOWESS) function. Note that the y-axis scales may be different for each sub-plot.

**Figure 6:**
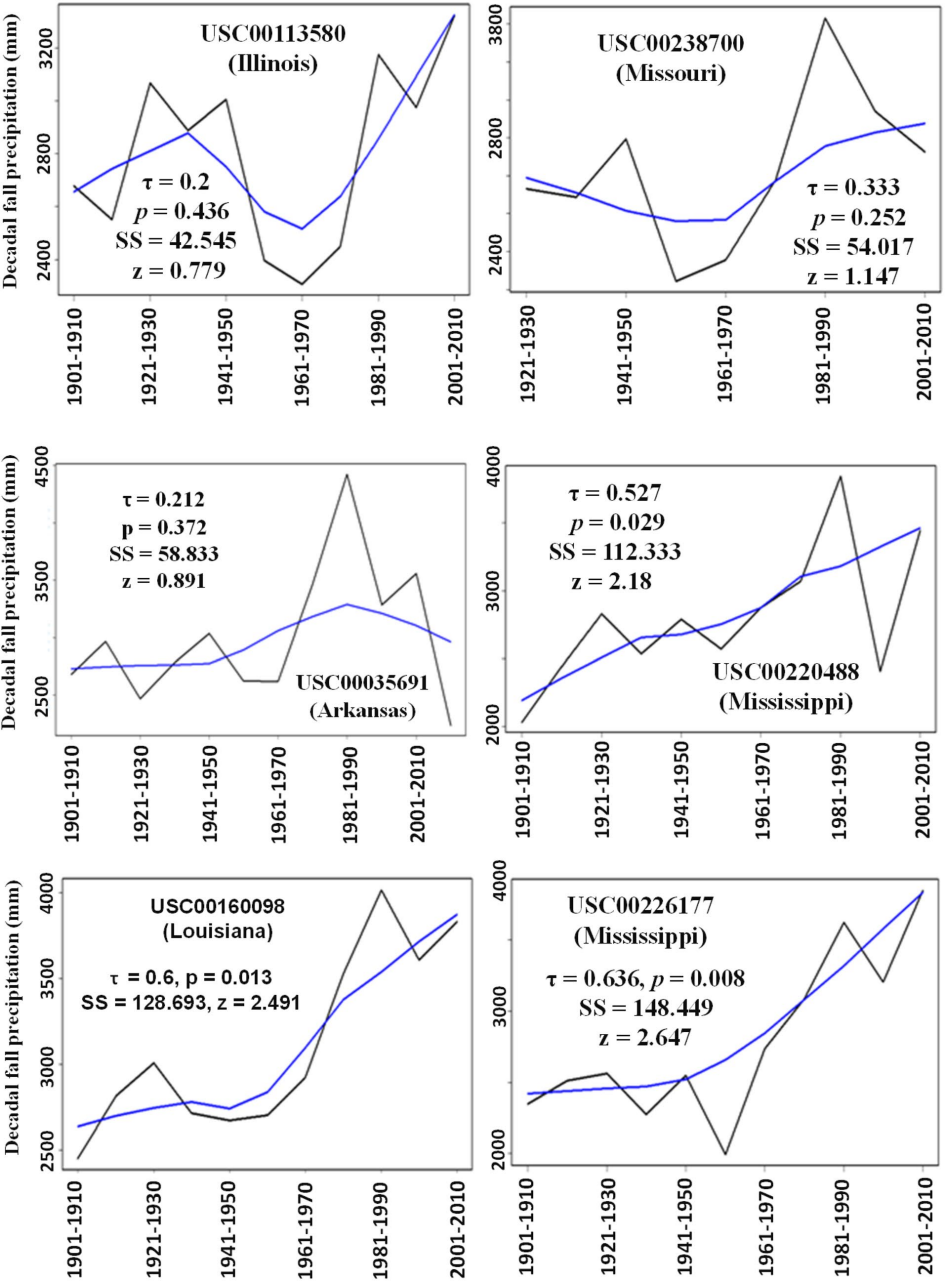
Amount of decadal precipitation in fall season for the six weather stations with Mann Kendall test results. The smoothing line is drawn using the Local Weighted Scatterplot Smoothing (LOWESS) function. Note that the y-axis scales may be different for each sub-plot.

Changes in total amounts of decadal spring and fall seasonal precipitations over the past 100 or more years for the six stations are shown in Figs. 5 and 6. There were very significant (*p* < 0.048) increasing trends with a profound rate (SS > 60) in total amounts of decadal spring precipitation for Stations USC00113580 and USC00238700, which are located in north LMRAV (Fig. 5). However, there were no significant trends in total amounts of decadal spring precipitation for the other four stations located at the center and south of the LMRAV (Fig. 5).

An opposite was true for the total amounts of decadal fall seasonal precipitations over the past 100 or more years. That is, there were significant (*p* < 0.013) and profound (SS > 120) increasing trends in total amounts of decadal fall seasonal precipitation for USC00226177 and USC00160098, which are close to the south coastal area of the LMRAV (Fig. 6). Additionally, there was one station (i.e., USC00220488) in the east center of the LMRAV with a strong (SS = 112) and significant (*p* < 0.03) increasing trend in total amount of decadal fall seasonal precipitation.

Table 2 reveals that there were two stations (USC00238700 and USC00035691) with the significant (*p* ≤ 0.016) and strong (SS > 79) increasing trends in total amounts of decadal winter precipitations over the past 100 years. The rate of the increasing trend of the USC00238700 (SS = 79.243 and located at the north LMRAV) was about one half of that of USC00035691 (SS = 190 and located at the west central LMRAV). Additionally, there was a decreasing trend in total amounts of decadal summer precipitation from four out of the six stations (Table 2) although this trend was not statistically significant.

### Linking precipitation trend to AMO

**Figure 7:**
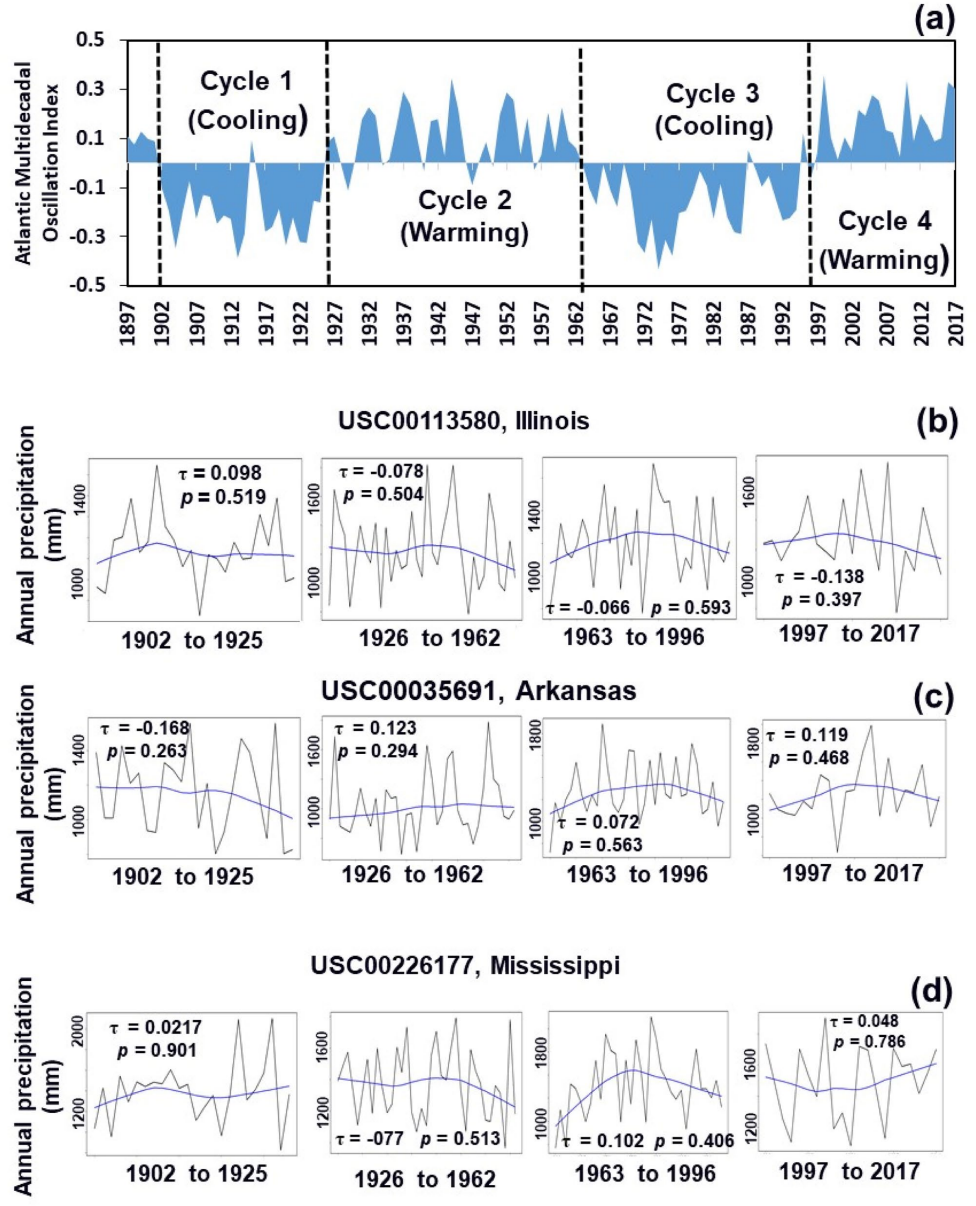
Annual precipitation trends for weather stations at Illinois (**b**), Arkansas (**c**), and Mississippi (**d**) in response to four Atlantic Multi-decadal Oscillation (AMO) cycles (**a**) from 1902 to 2017.

Comparisons of the annual precipitation trends from the three weather stations for each individual AMO cycle are shown in Fig. 7. The AMO is an index of the climate cycle that affects the sea surface temperature of the North Atlantic Ocean based on different modes on multi-decadal timescales. The negative AMO index is for a cooling cycle and the positive one is for a warming cycle^12^. The four AMO cycles used in this study were 1902 to 1925 (cooling cycle), 1926 to 1962 (warming cycle), 1963 to 1996 (cooling cycle), and 1997 to 2017 (warming cycle) (Fig. 7a). The three weather stations shown in the figure were USC00113580, USC00035691 and USC00226177, which are, respectively, located in the north, center, and south of the LMRAV (Fig. 1). There was no significant (*p* ≤ 0.05) precipitation trends for the three weather stations among the four individual AMO cycles (Fig. 7b, d). Our further analysis revealed that for the other three study sites, there was only one site (USC00238700 in Wappapello Dam, Missouri) at Cycle 3 from 1963 to 1996 with a significant increasing trend (*p* = 0.036, τ = 0.256, z = 2.094, and SS = 7.500 as shown in Table 3).

**Table 3:** Mann Kendall statistics of the annual precipitations for individual, two-consecutive, two cooling, and two warming AMO cycles for the six NOAA weather stations.

Parameter	USC00238700 in Wappapello Dam, Missouri	USC00113580 in Grand Chain Dam 53, Illinois	USC00035691 in Williams Junction, Arkansas	USC00220488 in Batesville, Mississippi	USC00160098 in Alexandria, Louisiana	USC00226177 in Natchez, Mississippi
**Individual cycle**						
Kendall's τ (Cycle 1)	− 0.071	− 0.098	− 0.168	0.107	0.192	0.022
Sen's slope (Cycle 1)	− 8.000	− 2.646	− 7.500	5.227	8.745	1.149
*p* value (Cycle 1)	0.902	0.519	0.264	0.469	0.197	0.901
z-score (Cycle 1)	− 0.124	− 0.645	− 1.118	0.724	1.290	0.124
Kendall's τ (Cycle 2)	− 0.012	− 0.078	0.123	0.092	− 0.146	− 0.077
Sen's slope (Cycle 2)	− 0.292	− 2.699	2.354	3.122	− 6.259	− 2.388
*p* value (Cycle 2)	0.927	0.505	0.295	0.432	0.209	0.513
z-score (Cycle 2)	− 0.092	− 0.667	1.047	0.785	− 1.256	− 0.654
Kendall's τ (Cycle 3)	0.256	0.066	0.072	− 0.038	0.225	0.102
Sen's slope (Cycle 3)	7.500	2.794	2.308	− 1.429	8.843	4.354
*p* value (Cycle 3)	0.036	0.594	0.563	0.767	0.064	0.406
z-score (Cycle 3)	2.094	0.534	0.579	− 0.297	1.854	0.830
Kendall's τ (Cycle 4)	− 0.138	− 0.138	0.119	− 0.236	0.124	0.048
Sen's slope (Cycle 4)	− 5.482	− 0.138	8.917	− 18.333	6.097	2.128
*p* value (Cycle 4)	0.398	0.398	0.468	0.172	0.450	0.786
z-score (Cycle 4)	− 0.846	− 0.846	0.725	− 1.367	0.755	0.272
**Two consecutive cycles**						
Kendall's τ (Cycles 1–2)	0.019	0.110	0.078	0.118	− 0.004	− 0.036
Sen's slope (Cycles 1–2)	0.436	2.333	1.667	2.333	− 0.045	− 0.698
*p* value (Cycles 1–2)	0.860	0.256	0.383	0.179	0.970	0.690
z-score (Cycles 1–2)	0.176	1.137	0.872	1.343	− 0.037	− 0.398
Kendall's τ (Cycles 2–3)	0.130	− 0.007	0.124	0.061	0.134	0.146
Sen's slope (Cycles 2–3)	2.647	− 0.231	1.667	1.429	2.842	2.629
*p* value (Cycles 2–3)	0.111	0.937	0.131	0.456	0.099	0.072
z-score (Cycles 2–3)	1.594	− 0.079	1.509	0.745	1.648	1.797
Kendall's τ (Cycles 3–4)	0.189	0.078	0.029	− 0.038	0.083	0.036
Sen's slope (Cycles 3–4)	4.384	1.746	0.645	− 1.429	0.376	1.139
*p* value (Cycles 3–4)	0.043	0.404	0.760	0.767	2.275	0.700
z-score (Cycles 3–4)	2.019	0.835	0.305	− 0.297	0.886	0.385
**Two warming cycles**						
Kendall's τ (Warming cycles)	0.131	− 0.098	0.094	0.116	0.038	0.104
Sen's slope (Warming cycles)	3.261	− 2.646	1.667	2.632	0.794	2.302
*p* value (Warming cycles)	0.149	0.519	0.304	0.198	0.677	0.254
z-score (Warming cycles)	1.443	− 0.645	1.027	1.289	0.416	1.141
**Two cooling cycles**						
Kendall's τ (Cooling cycles)	0.255	− 0.098	0.116	0.116	0.247	0.159
Sen's slope (Cooling cycles)	5.692	− 2.646	2.857	0.198	6.201	3.387
*p* value (Cooling cycles)	0.019	0.519	0.202	0.198	0.006	0.080
z-score (Cooling cycles)	2.344	− 0.645	1.275	1.289	2.724	1.751

Mann Kendall’s statistics of the annual precipitation trend analysis for the two consecutive AMO cycles (i.e., Cycles 1–2, Cycles 2–3, and Cycles 3–4), two warming AMO cycles, and two cooling AMO cycles are given in Table 3. The precipitation data of the two warming cycles (or the two cooling cycles) were obtained by simply added the time series annual precipitation data of the two cycles together. Table 3 shows that there was only one station (USC00238700) that had a significant (*p* = 0.043) increasing trend in the two consecutive Cycles 3–4 from 1963–2017, whereas there were two stations that had the very significant increasing precipitation trends (*p* = 0.019 for USC00238700 and *p* = 0.006 USC00160098) for the two cooling cycles combined (Table 3).

**Figure 8:**
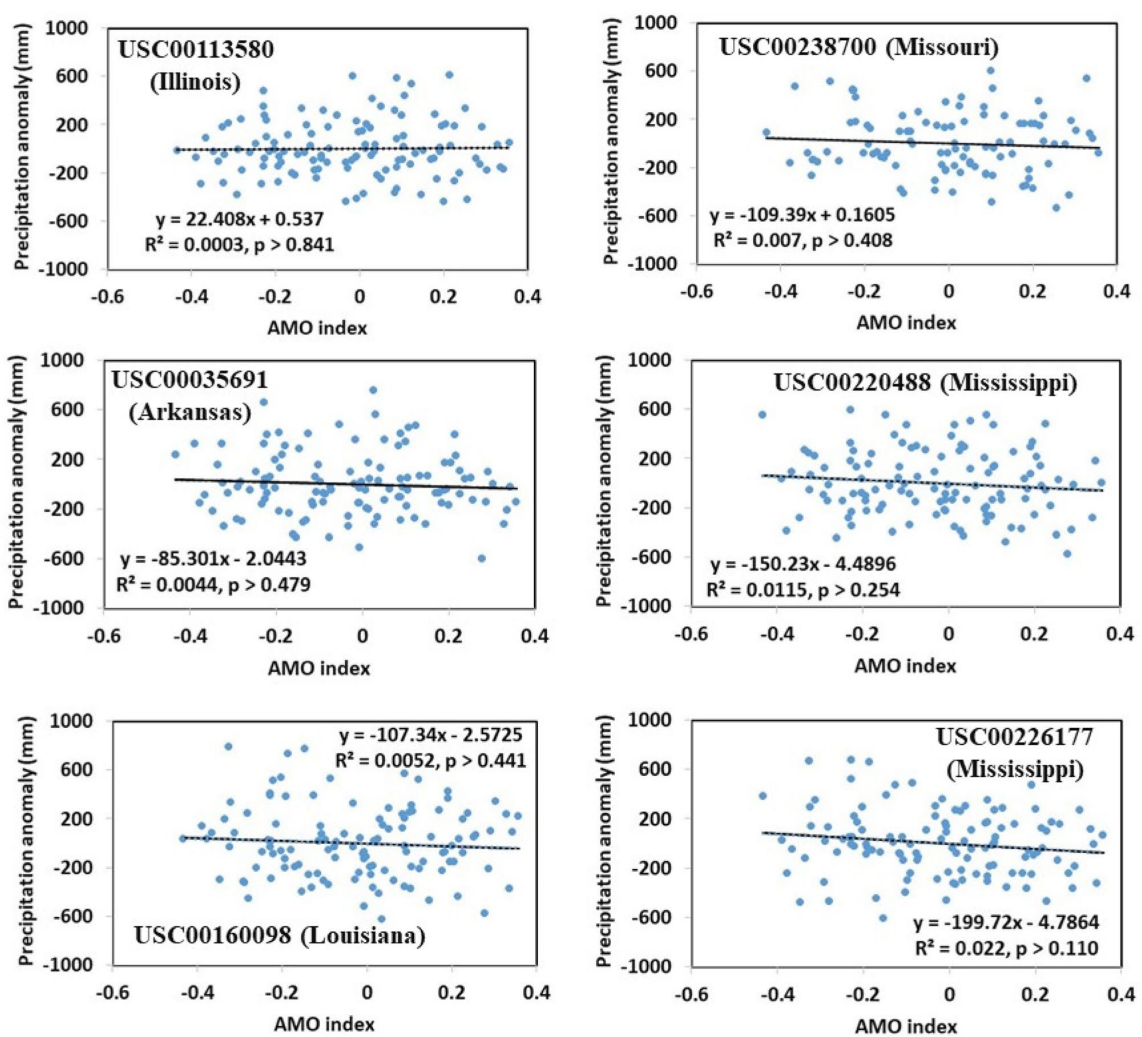
Correlations of the AMO index and the annual precipitation anomaly for all of the six study sites over the century-long period approximately from 1900 to 2017. The annual precipitation anomaly is the deviation of each annual precipitation from its annual average precipitation over the century-long period.

Plots of the AMO index against the precipitation anomaly over the century-long period (approximately 1900 to 2018) used in this study for all of the six stations are shown in Fig. 8. The annual precipitation anomaly was the deviation of each annual precipitation from its annual average precipitation over the century-long period. Figure 8 reveals that no correlations existed between the AMO index and the precipitation anomaly for all stations.

## Discussion

Results showed that a distinct spatial pattern of annual precipitation trends among the six stations in the forest lands from the south to north of the LMRAV over the past 100 or more years. More specifically, the forest lands close to the coastal area of the LMRAV had a significant increasing annual precipitation trend. This increasing trend became partially significant, i.e., significant in west central but not in east central LMRAV. Finally, the increasing trend was not significant in the north inland of the LMRAV. Recently, Abiy et al.^33^ investigated the rainfall trend and variability from 1906 to 2016 in the coastal area of Southeast Florida. These authors cited that the total wet season as well as the mean annual and total annual rainfalls have the increasing trends, supporting our findings. The coastal area of Southeast Florida and our coastal study sites are both within the Gulf of Mexico. Although our attempts to search literature reports on the similar studies at the center and north of Southeast US were not successful, we found a similar study from Asia^34^. Hamada et al.^34^ estimated the differences of rainfall characteristics between coastal and interior areas in central western Sumatra, Indonesia. These authors found that annual rainfall is generally abundant in the coastal region and sparser in the inland region, which is consistent with our findings. Although the exact reasons for this phenomenon remain to be investigated, a possible explanation would be that ocean is the largest contributor to atmospheric moisture and precipitation. Coastal areas receive more precipitation because there is much more evaporation from the oceans, which occurred due to the increase in air temperature as climate change. An increase in tropical cyclone intensity has also been observed, leading to an upward trend in North Atlantic hurricane activity since the 1970s^35^. As a result, the coastal areas had more rains than the inland areas within the LMRAV.

Analogous to the case of the annual precipitation trend, the number of the annual mean wet days in decadal scale had a similar temporal variability across space: a significant increasing trend near the coastal area of the LMRAV. It is apparent that the forest lands in the coastal area have become wetter as time elapsed over the past 100 plus years due to the same reasons as stated in the previous paragraph.

The significant increasing trends in decadal spring precipitation at the north inland of the LMRAV, in decadal fall precipitation near the coastal area of the LMRAV, and in decadal winter precipitation at the west-center and north-west area of the LMRAV over the past 100 years demonstrated the tempo-spatial seasonality of precipitation in the forest lands. Feng et al.^36^ reported the mean rainfall trend in the mesoscale convective system increases during spring in central USA. This finding is consistent with our result as the north inland of the LMRAV is located in central USA. However, the exact reasons for the increasing trends in different seasons at different locations remain to be investigated.

In spite of no statistically significant decreasing trends of precipitation in decadal summer season, four out of the six stations had negative τ and SS values, indicating a decreasing trend in decadal summer precipitation. Dyer and Mercer^4^ reported that rainfall amount is generally lower in warm season than in cool season at the LMRAV. Other researchers also predicted that the LMRAV will likely experience a general decrease in warm season precipitation over the coming decades^3,37,38^. A large portion of agricultural lands in the LMRAV relies on irrigation with groundwater resources for enhancing crop yields. Therefore, knowledge of the seasonal variations of precipitation in the LMRAV is crucial to water resources management and irrigation strategy development. Our findings of a wetter fall season in the coastal area and a potentially drier summer in the LMRAV over the past 100 years provide useful information to farmers and water resource managers for adapting climate change impacts.

High precipitation intensities were larger with shorter returning period and more frequent probability in the coastal area than in the inland of the LMRAV. This occurred because the sea surface temperature increased as climate change. The warmer temperature generated more water vapors from the ocean into the local atmosphere and increased the precipitation rate^35^. Therefore, the coastal area was more vulnerable to climate change in terms of precipitation.

Mixed impacts of the AMO cycles on annual precipitation trends were observed in the LMRAV. While most of such impacts were statistically not significant, there was one study site (USC00238700 in Wappapello Dam, Missouri) at the individual Cycle 3 (a cooling cycle from 1963 to 1996) with a very significant increasing annual precipitation trend. Oglesby et al.^39^ estimated the role of the AMO on medieval drought in North America. These authors argued that the AMO regulates the large-scale circulation to transport more precipitation during the cooling cycle and less precipitation during the warming cycle over the central and western U.S. They also cautioned that other factors such as El Niño-Southern Oscillation, Pacific Decadal Oscillation, local surface-atmosphere interactions, and soil moisture are also important in modulating precipitation. Our USC00238700 study site is located near the central U.S and therefore had an increasing precipitation trend during the cooling cycle. The other study sites that did not have significant precipitation trends for the individual cycles could occur due to the combined effects of AMO and other factors. Further study is therefore warranted to tackle on this issue in the LMRAV. Over the century-long period, the changes in AMO had no significant impacts on precipitation trends in the LMRAV since no correlations existed between the AMO index and the precipitation anomaly for all stations.

What is worth mentioning was the very significant increasing precipitation trends for the two stations (USC00160098 in Alexandria, LA and USC00226177 in Natchez, MS) near the coastal area of Gulf of Mexico when the two cooling cycles (from 1902 to 1925 and from 1963 to 1996) were combined. Results indicated that climate change affected the annual precipitation trends in the coastal area of the LMRAV during the cooling AMO cycles. This finding was consistent with those reported that the AMO transports more precipitations during the cooling cycle^39^.

## Methods

Six National Oceanic and Atmospheric Administration (NOAA) weather stations, namely the USC00238700 in Missouri, USC00113580 in Illinois, USC00035691 in Arkansas, USC00220488 in Mississippi, USC00160098 in Louisiana, and USC00226177 in Mississippi, were selected in this study (Fig. 1). They are located near the headwater areas of forest lands in the LMRAV. These weather stations were selected because they have continuous 100 or more years’ records of precipitation data. The daily precipitation data from the six stations were downloaded from the NOAAA website (https://www.ncdc.noaa.gov/cdo-web/datasets#GHCND). All of the stations except for USC00035691 have more than 100 years precipitation data from the NOAA website. For the USC00035691 station, the US-EPA BASINS Meterological Database was used to fill the data gaps from 1900 to 1964. Table 1 lists the station names, land uses, locations, and periods of precipitation records for the six selected weather stations.

The seasonal, annual, decadal, and AMO cyclic trends of precipitation for the six weather stations were assessed using Mann-Kendall trend analysis in R Statistics Platform (https://www.r-project.org), whereas the precipitation intensities with returning periods at given durations and time separations as well as the annual mean wet day frequency for every decade were analyzed using the commercially available HYDSTRA model (Kisters Inc.). The spring is from March to May, summer from June to August, fall from September to November, and winter from December to February.

The HYDSTRA Model (Version 12, Kisters Inc.) is a commercial software used to analyze time series data for climate, hydrology, and water quality. In this study, the HYSTATR function from the HYDSTRA Model was employed to analyze a variety of statistical parameters such as mean annual and maximum daily precipitation and mean wet day frequency that are related to a selected period of record of a precipitation site. The HYIFD function from the HYDSTRA model was utilized to scan precipitation data, extract a series of events for up to ten separate durations. An event is a precipitation intensity over a given duration and separation. The duration defines how long the precipitation intensity lasts, whereas the separation specifies a period of time that must elapse between the end of one event and the beginning of the next in order for them to be considered independent. A Log-Pearson type III analysis from the HYIFD function was then performed on these events to generate a set of average recurrent interval (e.g., time interval in years). The Log-Pearson Type III distribution is a statistical technique for fitting frequency distribution of time series data and is calculated using the general equation^40^ (https://streamflow.engr.oregonstate.edu/analysis/floodfreq/#log):

1 $$\log x = \overline{\log x} + + k\sigma_{\log x}$$

where x is the precipitation of some specified probability, $\overline{{\log x}}$ is the average of the log x precipitation values, k is a frequency factor, and $\sigma$ is the standard deviation of the log x values. In this study, the analysis was performed at annual scale with 24- and 72-h durations at a separation period of 24 h.

The Atlantic Multi-decadal Oscillation (AMO) cycles were used to relate the precipitation patterns in this study. AMO is an index of the climate cycle that affects the sea surface temperature of the North Atlantic Ocean based on different modes on multi-decadal timescales. The AMO index values were downloaded from https://www.esrl.noaa.gov/psd/data/timeseries/AMO/.

The Mann-Kendall trend analysis is a nonparametric trend test. The test does not require normally distributed data and is well suited for analyzing environmental, climate, and hydrological data^40^. The null hypothesis (H_o_) is that there is no trend, while the alternative hypothesis (H_a_) is that there is a trend. The test first ranks all observations by date (or time) order. Then the difference between each successive value is calculated, and the sum of the signs of those differences is evaluated as the Kendall sum statistic. The Mann-Kendall test statistic is calculated as^40–42^:

2 $$S = \mathop \sum \limits_{k = 1}^{n - 1} \mathop \sum \limits_{j = k + 1}^{n} sgn\left( {X_{j} - X_{k} } \right)$$

with

3 $$sgn = \left\{ {\begin{array}{*{20}l} {1\quad if\quad x > 0} \hfill \\ {0\quad if\quad x = 0 } \hfill \\ { - 1\quad if\quad x < 0} \hfill \\ \end{array} } \right.$$

The mean of S is zero and the variance is

4 $$\sigma = \frac{1}{18}\left\{ {n\left( {n - 1} \right)\left( {2n + 5} \right) - \mathop \sum \limits_{j = 1}^{m} t_{j} \left( {t_{j} - 1} \right)\left( {2t_{j} + 5} \right)} \right\}$$

where *m* is the number of the tied groups in the data set and t_j_ is the number of data points in the jth tied group. The Kendall’s S statistic is approximately normal distributed if the following Z-transformation is valid:

5 $$Z = \left\{ {\begin{array}{*{20}l} {\frac{s - 1}{\sigma }\quad if\quad S > 0} \hfill \\ {0\quad if\quad S = 0} \hfill \\ {\frac{s + 1}{\sigma }\quad if\quad S < 0} \hfill \\ \end{array} } \right.$$

The statistic S is closely related to Kendall's τ as:

6 $$\tau = \frac{S}{D}$$

with

7 $$D = \left[ {\frac{1}{2}n\left( {n - 1} \right) - \frac{1}{2}\mathop \sum \limits_{j = 1}^{p} t_{j} (t_{j} - 1)} \right]^{1/2} \left[ {\frac{1}{2}n\left( {n - 1} \right)} \right]^{1/2}$$

The Mann Kendall trend analysis is implemented with Kendall’s package in R-Statistics^31^.
